# Effect of Obesity on the Expression of Genes Associated with Severe Asthma—A Pilot Study

**DOI:** 10.3390/jcm12134398

**Published:** 2023-06-29

**Authors:** Marina Bantulà, Ebymar Arismendi, Valeria Tubita, Jordi Roca-Ferrer, Joaquim Mullol, Ana de Hollanda, Joaquín Sastre, Antonio Valero, Selene Baos, Lucía Cremades-Jimeno, Blanca Cárdaba, César Picado

**Affiliations:** 1 Institut d’Investigacions Biomèdiques August Pi i Sunyer (IDIBAPS), 08036 Barcelona, Spain; mbantulafonts@gmail.com (M.B.); earismen@clinic.cat (E.A.); v.tubita@ub.edu (V.T.); jrocaf@recerca.clinic.cat (J.R.-F.); jmullol@clinic.cat (J.M.); amdehol@clinic.cat (A.d.H.); valero@clinic.cat (A.V.); 2 Centro de Investigaciones Biomédicas en Red de Enfermedades Respiratorias (CIBERES), 28029 Madrid, Spain; jsastre@fjd.es (J.S.); bcardaba@quironsalud.es (B.C.); 3 Pulmonology Department, Hospital Clínic, Universitat de Barcelona, 08036 Barcelona, Spain; 4 Faculty of Medicine, Universitat de Barcelona, 08036 Barcelona, Spain; 5 Rhinology Unit & Smell Clinic, ENT Department, Hospital Clínic, Universitat de Barcelona, 08036 Barcelona, Spain; 6 Obesity Unit, Endocrinology and Nutrition Department, Hospital Clínic, Universitat de Barcelona, 08036 Barcelona, Spain; 7 Centro de Investigaciones Biomédicas en Red de Fisopatología de la Obesidad y Nutrición (CIBEROBN), 28029 Madrid, Spain; 8 Allergy Service, Instituto de Investigación Sanitaria Fundación Jiménez Díaz, Faculty of Medicine, Universidad Autónoma de Madrid, 28040 Madrid, Spain; 9 Allergy Department, Hospital Clínic, Universitat de Barcelona, 08036 Barcelona, Spain; 10 Immunology Department, Instituto de Investigación Sanitaria Fundación Jiménez Díaz, Universidad Autónoma de Madrid, 28040 Madrid, Spain; selenebmuniz@gmail.com (S.B.); lucia.cremades@quironsalud.es (L.C.-J.)

**Keywords:** asthma, obesity, biomarkers, gene expression, peripheral samples

## Abstract

Asthma is a complex condition resulting from the interaction of genes and environment. Obesity is a risk factor to develop asthma and contributes to poor response to asthma therapy and severity. The aim of the study was to evaluate the effect of obesity on the expression levels of genes previously associated with severe asthma. Three groups of subjects were studied: non-obese asthmatics (NOA), obese asthma patients (OA), and non-asthmatic obese subjects (O). Previously reported overexpressed (*IL-10*, *MSR1*, *PHLDA1*, *SERPINB2*, and *CD86*) and underexpressed genes (*CHI3L1*, *CPA3*, *IL-8*, and *PI3*) in severe asthma were analyzed by RT-qPCR in peripheral blood mononuclear cells (PBMCs). In the overexpressed genes, obesity significantly decreased the expression of *MSR1* and *PHLDA1* and had no effects on *CD86*, *IL-10*, and *SERPINB2*. In underexpressed genes, obesity did not affect *PI3*, *CHI3L1*, and *IL-8* and significantly reduced *CPA3* expression. The results of this study show that obesity should be included among the known factors that can contribute toward modifying the expression of genes associated with asthma and, in particular, severe asthma.

## 1. Introduction

Asthma is an inflammatory chronic respiratory disease clinically characterized by wheezing, shortness of breath, and cough associated with reversible airflow obstruction and airway hyperresponsiveness (AHR) [1]. Asthma displays a marked heterogeneity in etiology, clinical characteristics, and response to therapy. Asthma can be classified as allergic and non-allergic, eosinophilic and non-eosinophilic, and type 2 (T2)-high and T2-low, regarding the inflammatory profile [2,3]. T2-high endotype could be allergic or non-allergic depending on immunoglobulin E (IgE) levels and usually presents high numbers of eosinophils in the blood or sputum. Importantly, several studies indicate that asthma endotypes are also shared by other allergic diseases, such as chronic rhinosinusitis with nasal polyps (CRSwNP). Patients with asthma and CRSwNP are frequently characterized by a predominant T2 airway inflammation, affecting the lower and upper airways, respectively [4]. The T2-low endotype is counterbalanced by a high number of neutrophils in bronchoalveolar fluid (BALF) and sputum and high clinical severity as seen in late onset non-allergic asthma or obesity-associated asthma [3].

Numerous studies have investigated the role of genes involved in the regulation of inflammatory responses in severe asthma. Genome-wide association studies have identified many common variant loci associated with asthma susceptibility, as well as the genetics underlying moderate-to-severe asthma risk [5,6]. However, genetic variants associated with moderate-to-severe asthma overlap significantly, reflecting that the genetic contribution to asthma severity is most probably modest in comparison with the more relevant role played by environmental factors such as smoking exposure [7,8] and rhinovirus infections early in life [9].

Obesity is a systemic pathophysiological state that, via poorly understood mechanisms, changes the structure of immune responses resulting in a systemic low-grade inflammatory disease [2]. Obesity-related inflammation begins in visceral adipose tissue, and the major cells responsible for such inflammation are macrophages, B and T lymphocytes, and neutrophils. These cells express a large amount of pro-inflammatory mediators, such as interleukin (IL)-1β, IL-6, IL-8, and tumor necrosis factor alpha (TNF-α) [2].

Excess body weight has been associated with an increased risk of multimorbidities, such as asthma [2]. Obesity-associated asthma (OA) often has a high disease burden, despite the use of high-dose inhaled corticosteroids (ICS). In contrast to asthma patients with normal weight, the clinical efficacy of inhaled corticosteroid therapy in OA patients is often relatively low [10]; however, the mechanism by which obesity alters the pathology and treatment response in asthma is only partially known. The reduced response to corticosteroids in OA patients with respect to non-OA, which recovers after weight loss, can contribute towards explaining the poor response of obese asthma to this therapy [11].

Weight loss is an effective treatment for severe obesity and obesity-related co-morbidities such as asthma [2]. However, the mechanism by which weight loss improves asthma is only partially known [2,11,12].

Despite the accumulated evidence showing that obesity is a risk factor for developing severe asthma, the possibility that this effect could be mediated, at least in part, via modification of the expression of genes associated with asthma severity has been poorly studied. Some studies analyzed microRNA expression and found higher levels of hsa-miR-155-5p and 223-5p in moderate asthmatics and obese subjects compared to healthy controls [13,14].

In previous studies, we reported a set of genes that were overexpressed (*IL-10*, *MSR1*, *PHLDA1*, *SERPINB2*, and *CD86*) or underexpressed (*CHI3L1*, *CPA3*, *IL-8*, and *PI3*) in severe asthma patients compared with control subjects [15,16].

Given the proved impact of obesity on asthma severity, it becomes rational to examine the relationship between obesity and the expression of genes associated with severity. The study aimed to evaluate the effect of obesity on the expression levels of these genes previously associated with severe asthma.

## 2. Materials and Methods

### 2.1. Participants

We recruited 47 participants: 22 patients with OA [Body mass index (BMI) ≥ 30 kg/m^2^], 12 non-obese asthma (NOA) patients (BMI < 25 kg/m^2^), and 13 obese non-asthma subjects (O). The following criteria were used to select asthmatic patients: (1) a clinical history of asthma and (2) either bronchodilator responsiveness (>12% and 200 mL improvement in forced expiratory volume in 1 s (FEV_1_) after 400 µg salbutamol metered-dose inhaler) or positive response to a methacholine bronchoprovocation test. None of the subjects had received systemic corticosteroids for one month or longer prior to evaluation. Forced spirometry was performed according to ERS/ATS standards [17]. Obese subjects had no history of asthma or wheezing, had no other chronic respiratory disease, and had never smoked.

The collaborating endocrinologist presented the objective of the study to the subjects with obesity during the pre-surgery evaluation; those who initially agreed to participate were referred to the Respiratory Department for further studies. All subjects enrolled were provided with an information sheet describing the research objectives and requirements for participation. The study was approved by the Ethics Committee of the Hospital Clinic Barcelona (2018/4015). All subjects provided signed informed consent to participate in the study. An observational study design was used to assess the effect of weight on the expression of selected genes.

### 2.2. Blood Collection and PBMCs Isolation

Whole blood (10 mL) was collected from each patient via venipuncture into a vacutainer tube containing an anticoagulant (EDTAK_2_). Peripheral blood mononuclear cells (PBMCs) were isolated using Lymphoprep^TM^ (Stem Cell TM, Bernburg, Germany) following the manufacturer’s instructions. Whole blood was diluted 1:1 with a balanced salt solution, and this was then layered on top of the Lymphoprep solution in a 50 mL conical tube and centrifuged at 800× *g* for 20 min at room temperature. After centrifugation, the white ring containing mononuclear cells was transferred to a new tube and washed twice with a balanced salt solution.

### 2.3. RNA Extraction and cDNA Synthesis

Total RNA was isolated from PBMCs using the TRIzol reagent (Life Technologies, Paisley, UK) according to the manufacturer’s protocol. Total mRNA concentration was measured at 260 nm, and purity was assessed from the 260/280 nm absorbance ratio. One µg of mRNA was converted into cDNA using the High-Capacity cDNA Reverse Transcription Kit according to the manufacturer’s instructions (Thermo Fisher, Vilnius, Lithuania). Samples were incubated for 10 min at 25 °C, 120 min at 37 °C, and 5 min at 85 °C. Final cDNA products were diluted 10-fold before use in qPCR.

### 2.4. Real-Time qPCR

The expression of nine previously selected genes (Table 1) was analyzed with real-time qPCR. qPCR experiments were carried out using 100 ng of cDNA in the Viia7 Real-Time PCR system (Applied Biosystems, Carlsbad, USA) following the manufacturer’s guidelines. The qPCR reaction consisted of 0.5 µL of TaqMan gene Expression Assay (20×), 5 µL of TaqMan Universal PCR Master Mix (2×), 2.5 µL of RNase- free water, and 2 µL of cDNA in a total volume of 10 µL. The thermal cycler was set to 95 °C for 20 min, followed by 40 reaction cycles of 1 s at 95 °C and 20 s at 60 °C. Specific mRNA expression from each gene was analyzed in duplicate and normalized against 18S rRNA and GAPDH genes. Data were analyzed using the ΔΔCt (double delta Ct) method and represented as Fold Change (FC) after normalizing against NOA participants’ mRNA levels.

### 2.5. Statistical Analysis

Clinical and experimental data were reported as the median and interquartile range. Comparisons of gene expression levels between groups were performed using the Kruskal–Wallis H test for nonparametric data followed by the post hoc Dunn’s multiple comparisons test. Differences between two groups were analyzed using the Mann–Whitney U test for non-parametric data. All analyses were performed using GraphPad Prism version 8.4 for Windows, (GraphPad Software, La Jolla, CA, USA). Statistical significance was defined as a *p*-value < 0.05.

## 3. Results

The demographic and clinical characteristics of the participants are depicted in Table 2. Following standard recommendations, the asthma severity level was established according to the number of antiasthma drugs (bronchodilators and ICS) and ICS dosage used to control the disease. Asthmatic patients with elevated specific IgE against one or more allergens were classified as atopic. Finally, patients were divided into two groups of more or less than 300 blood eosinophils/µL.

### 3.1. Effects of Obesity on Overexpressed Genes

**Figure 1:**
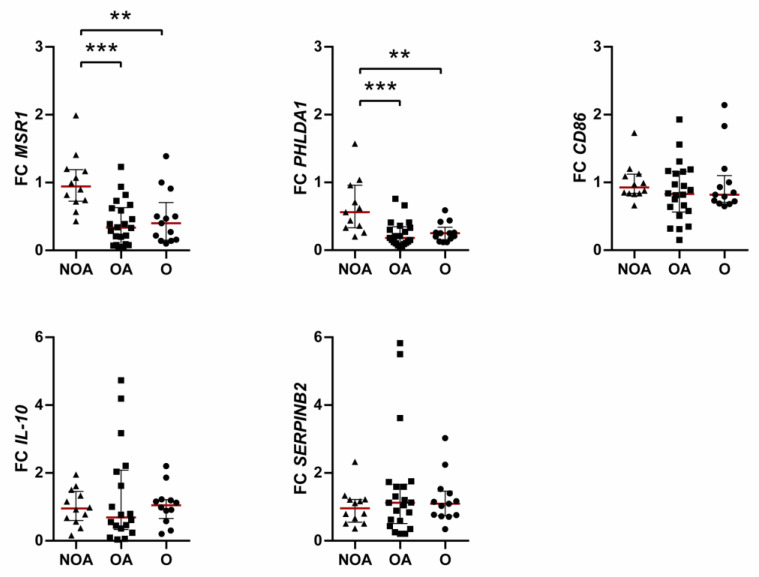
Differential expression of overexpressed genes among clinical phenotypes. Data presented as individual values and as medians (red line) with 25th–75th percentile. FC, fold change; NOA, non-obese asthmatics; OA, obese asthmatics; O, obese subjects. ** *p* ≤ 0.01, *** *p* ≤ 0.001; Kruskal–Wallis followed by Dunn’s multiple comparisons test.

Obesity exerts contrasting effects on overexpressed genes, significantly decreasing the expression of *MSR1* and *PHLDA1* but with no statistically significant effects on *CD86*, *IL-10*, and *SERPINB2* (Figure 1). However, in patients with blood eosinophils count higher than 300 cells/µL, *IL-10* expression was decreased, compared with low eosinophilic subjects (*p* = 0.0275). Moreover, in severe asthmatics with and without obesity, *MSR1* expression was higher compared with mild asthmatics (*p* = 0.0394). No differences were found regarding atopy status or gender.

### 3.2. Effects of Obesity on Underexpressed Genes

**Figure 2:**
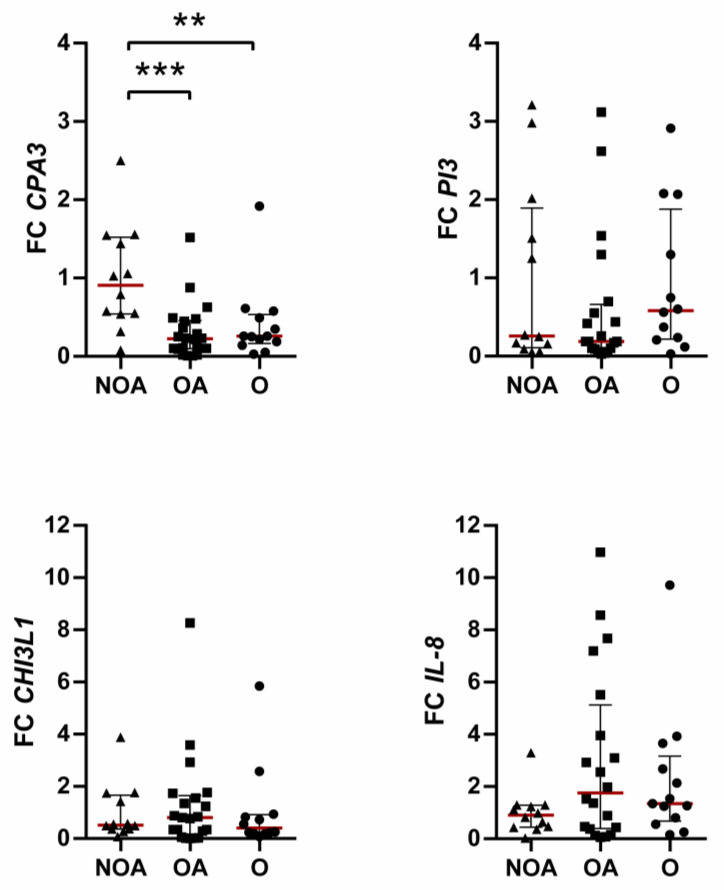
Differential expression in underexpressed genes among clinical phenotypes. Data presented as individual values and as medians (red line) with 25th–75th percentile. FC, fold change; NOA, non-obese asthmatics; OA, obese asthmatics; O, obese subjects. ** *p* ≤ 0.01, *** *p* ≤ 0.001; Kruskal–Wallis followed by Dunn’s multiple comparisons test.

Similarly, in underexpressed genes, obesity (OA and O) significantly reduced *CPA3* expression but did not affect *PI3*, *CHI3L1*, and *IL-8* expression (Figure 2). Stratifying patients according to blood eosinophils, asthma severity, atopy status, and gender presented no gene expression differences between groups.

## 4. Discussion

This research examined the changes induced by obesity in the expression of genes previously reported to be linked with severe asthma. Several studies corroborated the existence of an excess risk of developing asthma in obese subjects, with a greater risk in females with respect to males, presenting a late-onset, non-atopic asthma [18,19,20,21,22]. This fact that is reflected in our study population, where more than 80% of the participants are women. The inequality between the number of participants of both genders could be the reason why we have not found gender differences in the expression of these genes. In our study, 67% of non-obese asthmatic participants were atopic and presented high blood eosinophils. In contrast, only 41% of obese asthmatic participants were atopic, and 32% presented high blood eosinophils. However, gene expression did not change according to atopy status.

IL-10 is a T helper (Th)2-type cytokine that is produced by a wide range of immunological cell types, including monocytes/macrophages, different lymphocyte types (Th1, Th2, cytotoxic, and B), dendritic cells, and mast cells. IL-10 is also a potent inhibitor of some pro-inflammatory cytokines such as TNF-α and IL-6 [23,24]. In a previous study, we found upregulation of the *IL-10* gene in peripheral blood cells of severe asthma patients [15,16]. In contrast, an inverse association between asthma severity and IL-10 concentration in BALF was reported by Borish et al. [25]. The absence of IL-10 in asthma causes the continued secretion of pro-inflammatory cytokines such as IL-6, IL-5, IL-4, TNF-α, GM-CSF, and IL-1, which can contribute to enhancing asthmatic airway inflammation [23]. Differences in the methods used between studies (PBMC vs. BAL; qPCR vs. ELISA) can account for the discrepant findings between our studies and those of Borish et al. [25]. In the present study, we found that OA subjects (most of them women) appear to be associated with an increased expression of *IL-10*; however, the difference did not reach statistical significance. However, *IL-10* expression was decreased in patients with high blood eosinophil count (≥300 cells/µL) compared with non-eosinophilic participants. Previous studies reported upregulated *IL-10* expression in white adipose tissue associated with elevated circulating levels of IL-10 in obese women compared with non-obese women. These findings were not observed in men [26,27,28]. However, how this increased IL-10 production associated with obesity impacts the inflammatory process underlying OA remains to be elucidated.

SERPINB2 or plasminogen activator inhibitor-2 (PAI-2) is an inhibitor of the urokinase plasminogen activator (uPA). *SERPINB2* is expressed in a large number of cell types, including immune cells, and is involved in various cellular functions such as cell survival, migration and differentiation, inflammation, immunity, and extracellular matrix remodeling [29,30]. *SERPINB2* has been implicated in the pathogenesis of various diseases, including malignancies and immune-associated inflammatory diseases [29,30,31], and has been included among the genes for the identification of asthma patients with Th2-high immunity [32]. To the best of our knowledge, the impact of obesity on *SERPINB2* expression has not been reported; we did not find any significant change in *SERPINB2* expression in obese subjects. 

Macrophages (M) are the most abundant immune cell type in the airways. Macrophages can differentiate into M1 (pro-inflammatory) or M2 (anti-inflammatory) subtypes. M1 macrophages are induced by Th1 stimulation via IFN-γ, while M2 macrophages are induced by Th2 stimulation via IL-4 and IL-13 [33]. Macrophages with an M2 phenotype (expressing high levels of CD206) are increased in the airways of asthma patients [34]. Indeed, hsa-miR-155-5p expression is increased in CD4+ T cells of asthmatics compared to non-asthmatics and positively associated with Th2 cytokine profile [14]. In contrast, circulating M1 macrophages (expressing high levels of CD86) are decreased in moderate and severe asthmatic children [35]. The low-grade inflammatory response usually found in obesity increases monocyte recruitment and activation from the circulation and facilitates M1 macrophage accumulation instead of converting monocytes to M2 macrophages [36]. Hsa-miR-155-5p has been reported to be overexpressed in obese adipose tissue macrophages exosomes [14]. In keeping with these observations, we found a trend toward decreased *CD86* expression in both asthma and obese subjects but with no evidence of additive effects.

Macrophage scavenger receptor 1 (MSR1), also known as scavenger receptor-A (SR-A), induces immune protection by limiting M polarization towards the pro-inflammatory M1 phenotype and, therefore, decreasing the secretion of pro-inflammatory cytokines (IL-1b, IL-6, and TNF-α) [37]. Although initially described in macrophages, *MSR1* is also present in numerous cells such as PBMCs, lung epithelial cells, endothelial cells, and cells of the neurological system [37,38,39,40,41]. In previous studies, our group reported that *MSR1* is overexpressed in the PBMCs of patients with severe asthma, particularly in those with a non-allergic phenotype [15,16,41,42,43] and in patients with chronic obstructive pulmonary disease (COPD) [43]. In the present study, we also found higher *MSR1* expression in severe asthmatics with and without obesity. Moreover, highly expressed *MSR1* was found in the four PBMC subpopulations evaluated (CD4+ and CD8+ lymphocytes, B lymphocytes, and monocytes) [43]. Recent studies support the notion that *MSR1* is involved in inflammatory responses in obesity [44,45]. *MSR1* expression in adipocytes is increased in obese subjects compared with non-obese controls [45], and blocking *MSR1* reduces foamy macrophage formation and the release of inflammatory cytokines such as TNF-ɑ [44]. Taken together, these observations suggest that, by targeting *MSR1*, it would be possible to reduce lipid-induced inflammation [44]. Interestingly, in our study, the presence of obesity significantly downregulates overexpressed *MSR1* in asthma patients. How this effect can contribute to the association of obesity with asthma severity remains to be investigated.

Pleckstrin homology-like domain, family A, member 1 (PHLDA1), which is also called T-cell death-associated gene 51 (TDAG51), has been involved in several biological processes, including cell proliferation, apoptosis, and differentiation and in sepsis, ulcerative colitis, and some human malignancies [46,47,48,49]. A recent study also found that *PHLDA1* is significantly highly expressed in patients with COPD [50]. Regarding the relationship between *PHLDA1* and obesity, it was reported that *PHLDA1* is expressed in preadipocytes and is downregulated during adipogenesis. Moreover, *PHLDA1* expression has been found inversely correlated with fatty liver in several mouse models of hepatic steatosis [51,52]. In keeping with these observations, we found that obesity in OA and O subjects without asthma was associated with a significant reduction in *PHDLA1* gene expression in comparison with NOA.

Chitinase-3-like protein 1 (CHI3L1), also named YKL-40, is one of several known human chitinases [53]. Chitinases probably contribute to the defense mechanism against chitin-containing parasites and fungi [53]. Elevated CHI3L1 levels have been found in serum and BAL fluid in asthma patients compared with healthy controls [12,53]. In addition, serum CHI3L1 levels correlate with poor asthma control, subepithelial membrane thickness, FEV_1_ decline, and severity of airway obstruction [12,53,54,55,56].

High serum levels of CHI3L1 correlating with BMI have been reported in obese subjects [12,57,58]. The study of the potential additive effects of obesity and asthma on serum CHI3L1 levels yielded contrasting results, with some studies reporting additive effects [59] that were not found in others [12,60]. In the present study, obesity did not modify the expression of the *CHI3L1* gene, which suggests that the previously reported effects of obesity on CHI3L1 protein serum levels should take place at translational or post-translational levels.

*PI3* (peptidase inhibitor 3 skin-derived) encodes a serine protease inhibitor (elafin), which is synthesized and secreted by numerous cells (monocytes, neutrophils, and airway epithelial cells) at the site of injury to modulate the potentially deleterious effects of excessively released proteases (trypsin, chymotrypsin, and elastase) in injured tissues [61,62]. PI3 synthesis and secretion are induced by cytokines such as TNF and IL-1β [61,62]. *PI3* is upregulated in adipose tissue in proportion to the degree of inflammation, a finding that supports a role for *PI3* in dampening the adipose-related inflammatory process [63]. We observed increased *PI3* expression in obese subjects with respect to non-obese subjects; however, the difference did not reach statistical significance. A single study examined the relationship between PI3 and asthma, finding that plasma PI3 levels were significantly lower in patients than in healthy controls [64]. The mechanism responsible for this finding and its potential role in asthma are currently unknown.

IL-8 is a potent chemoattractant that regulates the activation and migration of neutrophils to the inflammation site through the high-affinity CXC motif chemokine receptor 2 (CXCR2) expressed on the surface of neutrophils. Increased levels of IL-8 in sputum have been reported in patients with neutrophilic asthma, which correlated with sputum neutrophil counts and increased in uncontrolled disease [65,66,67]. Studies have shown that neutrophils are also associated with allergic inflammation. Airway exposure to allergenic extracts recruits neutrophils to the airways and increases IL-8 levels in human subjects with asthma or seasonal allergic rhinitis [68,69].

Circulating IL-8 levels in exhaled air are elevated in obese subjects and associated with obesity-related parameters such as BMI [70]. Similarly, IL-8 and neutrophil numbers were higher in induced sputum in obese subjects compared with non-obese controls [71]. In keeping with these observations, we found that the *IL-8* gene was overexpressed in obese subjects, particularly in OA patients. *IL-8* was overexpressed in OA with respect to NOA subjects; however, the difference was not statistically significant, probably due to the high variability, especially in the OA participants.

Metalloprotease carboxypeptidase A3 (CPA3) is one of the most abundant mast cell proteases [72] and appears to play important inflammatory and remodeling roles in asthma and COPD [73,74,75]. Increased *CPA3* expression has been found in airway mast cells of asthma patients with the Th2-high phenotype [76]. In addition, *CPA3* gene expression correlates with blood eosinophilia, eosinophilic airway inflammation [77], and asthma severity [73].

The role of mast cells in obesity and obesity-related pathologies is a matter of controversy. Using animal models of obesity, some studies concluded that mast cells do not play any relevant role in the pathogenesis of obesity [74]. However, recent studies suggest that mast cells may be involved in the mechanisms used by mammalians to adapt their bodies to cold. Brown and beige adipose tissues generate heat by uncoupling oxidative respiration in mitochondria; mast cells responded to cold releasing IL-4 [75]. Interestingly, the cell marker CPA3 predicts the expression of the uncoupling protein (UCP1), which is involved in the mechanism that favors heat generation [75]. These observations suggest that, in contrast to previous opinions, mast cells may play an important role in adipose tissue regulation and that *CPA3* expression can be used as a marker of their activity [75]. In our study, for the first time, we found that obesity significantly decreases *CPA3* expression in PBMCs of asthmatic and non-asthmatic obese subjects. The significance and impact of this observation in the pathogenesis of the obese asthma phenotype remain to be elucidated.

The major drawback of our study is the relatively small number of subjects studied cross-sectionally. Results need to be examined in a larger cohort and the impact of asthma inflammatory endotypes (T2 or non-T2) on gene expression should also be considered in future studies. The specific effects of obesity on asthma-related genes require verification in patients submitted to therapies aimed at reducing weight.

## 5. Conclusions

In summary, asthma is one of the most common lung diseases in humans, affecting children and adults. The basis of asthma is an interplay between genetics and the environment. Several genes contribute to disease risk, and others regulate asthma severity. Gene–environment interactions can contribute to the development of asthma and increase its severity. The results of the present study show that the expression of *MSR1*, *PHLDA1*, and *CPA3*, previously reported associated with severe asthma, are modified by the presence of obesity. Therefore, obesity should be included among the known factors that can contribute to modifying the expression of genes associated with asthma and, in particular, severe asthma.

## Figures and Tables

**Table 1:** List of nine genes analyzed and two reference genes.

Gene Symbol	Gene Name	Detector
*CD86*	CD86 molecule	Hs01567026_m1
*CHI3L1*	Chitinase 3-like 1 (cartilage glycoprotein-39)	Hs00609691_m1
*CPA3*	Carboxypeptidase A3 (mast cell)	Hs00157019_m1
*IL-8*	Interleukin 8	Hs00174103_m1
*IL-10*	Interleukin 10	Hs00961622_m1
*MSR1*	Macrophage scavenger receptor 1	Hs00234007_m1
*PHLDA1*	Pleckstrin homology-like domain, family A, member 1	Hs00705810_s1
*PI3*	Peptidase inhibitor 3, skin-derived	Hs00160066_m1
*SERPINB2*	Serpin peptidase inhibitor, clade B, member 2	Hs01010736_m1
*18S*	Eukaryotic 18S rRNA	Hs99999901_s1
*GAPDH*	Glyceraldehyde-3-phosphate dehydrogenase	Hs02758991_g1

**Table 2:** Baseline demographic and clinical data of the study population.

	NOA (*n* = 12)	OA (*n* = 22)	O (*n* = 13)
Age, years	54.5 (41.5–59.5)	57.0 (51.0–61.5)	47.0 (45.5–61.5)
Female, *n* (%)	10 (83.3)	18 (81.8)	11 (84.6)
BMI, kg/m^2^	23.2 (22.0–25.0)	38.0 (35.1–45.0) *	42.2 (38.4–47.9) *
Mild asthma, *n* (%)	0 (0)	4 (18.2)	N/A
Moderate asthma, *n* (%)	5 (41.7)	5 (22.7)	N/A
Severe asthma, *n* (%)	7 (58.3)	13 (59.1)	N/A
FVC, % predicted	128.0 (112.3–138.5)	110.5 (94.5–119.3) *	118.5 (105.5–125.8)
FEV_1_, % predicted	79.0 (69.8–98.5)	79.0 (61.5–93.5)	90.0 (87.0–100.8)
FEV_1_/FVC	65.5 (57.0–74.8)	76.0 (66.5–80.5)	80.5 (75.0–82.8) *
Use of ICS ^§^, *n* (%)	10 (83.3)	16 (72.7)	N/A
Atopia, *n* (%)	8 (66.7)	9 (40.9)	N/A
Serum total IgE, kU/L	122.0 (42.3–409.0)	63.7 (14.4–149.0)	50.0 (17.9–112.5)
BEC, %	4.8 (3.3–6.6)	3.3 (2.3–4.9)	2.8 (1.4–3.5) *
BEC, cells/µL	300 (200–500)	200 (200–400)	200 (100–300)
BEC ≥ 300 cells/µL, *n* (%)	8 (66.7)	7 (31.8)	3 (23.1)

Data presented as medians (25th–75th percentile). NOA, non-obese asthmatics; OA, obese asthmatics; O, obese subjects; BEC, blood eosinophil count; BMI, body mass index; FVC, forced vital capacity; FEV_1_, forced expiratory volume in 1 s; ICS, inhaled corticosteroids; IgE, immunoglobulin E; N/A, no applicable. * *p* < 0.05, compared with NOA; Kruskal–Wallis H followed by Dunn’s multiple comparisons test. ^§^ For NOA and OA patients who received ICS, the mean ± SD of the ICS dose in budesonide equivalents was 680.0 ± 518.5 and 1356.2 ± 913.7 µg/day, respectively.

## Data Availability

Data generated or analyzed during this study are included in this published article or available from the corresponding author on request. All analyzed datasets were sourced from the authors.
